# Water-soluble aluminium phthalocyanine–polymer conjugates for PDT: photodynamic activities and pharmacokinetics in tumour-bearing mice

**DOI:** 10.1038/sj.bjc.6690557

**Published:** 1999-07

**Authors:** N Brasseur, R Ouellet, C La Madeleine, J E van Lier

**Affiliations:** MRC Group in the Radiation Sciences, Faculty of Medicine, Université de Sherbrooke, Sherbrooke, Québec, J1H 5N4, Canada

**Keywords:** Colo-26 tumour, EMT-6 tumour, aluminium phthalocyanine, photodynamic therapy, polyethyleneglycol, polyvinylalcohol

## Abstract

The potential use of unsubstituted aluminium phthalocyanine (AlClPc) as a sensitizer for photodynamic therapy (PDT) of cancer has not been fully exploited in spite of its higher efficiency as compared to the sulphonated derivatives. This is largely due to the strong hydrophobic character of AlClPc which renders the material difficult to formulate for in vivo administration. We prepared two water-soluble derivatives of AlClPc by axial coordination of polyethyleneglycol (PEG, MW 2000) or polyvinylalcohol (PVA, MW 13 000–23 000) to the central aluminium ion. Their photodynamic activities were evaluated in vitro against the EMT-6 mouse mammary tumour cells and in vivo against the EMT-6 and the colon carcinoma Colo-26 tumours implanted intradermally in Balb/c mice. Pharmacokinetics were studied in the EMT-6 tumour-bearing mice. After 1 h incubation, the light dose required to kill 90% of cells (LD_90_) was at least three times less for AlClPc (Cremophor emulsion) as compared to AlPc–PEG and AlPc–PVA, while after 24 h incubation all three preparations were highly phototoxic. All three dye preparations induced complete EMT-6 tumour regression in 75–100% of animals at a low drug dose (0.25 μmol kg^−1^) following PDT (400 J cm^−2^, 650–700 nm) at 24 h pi. Complete tumour regression in the Colo-26 tumour model was obtained in 30% of mice at a dose of 2 μmol kg^−1^. In the non-cured animals, AlPc–PVA induced the most significant tumour growth delay. This dye showed a prolonged plasma half-life (6.8 h) as compared to AlClPc (2.6 h) and AlPc–PEG (23 min), lower retention by liver and spleen and higher tumour-to-skin and tumour-to-muscle ratios. Our data demonstrate that addition of hydrophilic axial ligands to AlPc, while modifying in vitro and in vivo kinetics, does not reduce the PDT efficiency of the parent molecule. Moreover, in the case of the polyvinylalcohol derivative, axial coordination confers advantageous pharmacokinetics to AlPc, which makes this photosensitizer a valuable, water soluble candidate drug for clinical PDT of cancer. © 1999 Cancer Research Campaign


*British Journal of Cancer* (1999) **80**(10), 1533–1541
© 1999 Cancer Research Campaign
Article no. bjoc.1999.0557

### Water-soluble aluminium phthalocyanine–polymer conjugates for PDT: photodynamic activities and pharmacokinetics in tumour-bearing mice

**N Brasseur, R Ouellet, C La Madeleine and JE van Lier**

MRC Group in the Radiation Sciences, Faculty of Medicine, Université de Sherbrooke, Sherbrooke, Québec, J1H 5N4, Canada

**Summary** The potential use of unsubstituted aluminium phthalocyanine (AlClPc) as a sensitizer for photodynamic therapy (PDT) of cancer has not been fully exploited in spite of its higher efficiency as compared to the sulphonated derivatives. This is largely due to the strong hydrophobic character of AlClPc which renders the material difficult to formulate for in vivo administration. We prepared two water-soluble derivatives of AlClPc by axial coordination of polyethyleneglycol (PEG, MW 2000) or polyvinylalcohol (PVA, MW 13 000–23 000) to the central aluminium ion. Their photodynamic activities were evaluated in vitro against the EMT-6 mouse mammary tumour cells and in vivo against the EMT-6 and the colon carcinoma Colo-26 tumours implanted intradermally in Balb/c mice. Pharmacokinetics were studied in the EMT-6 tumour-bearing mice. After 1 h incubation, the light dose required to kill 90% of cells ($LD_{90}$) was at least three times less for AlClPc (Cremophor emulsion) as compared to AlPc–PEG and AlPc–PVA, while after 24 h incubation all three preparations were highly phototoxic. All three dye preparations induced complete EMT-6 tumour regression in 75–100% of animals at a low drug dose (0.25 µmol $kg^{-1}$) following PDT (400 J $cm^{-2}$, 650–700 nm) at 24 h pi. Complete tumour regression in the Colo-26 tumour model was obtained in 30% of mice at a dose of 2 µmol $kg^{-1}$. In the non-cured animals, AlPc–PVA induced the most significant tumour growth delay. This dye showed a prolonged plasma half-life (6.8 h) as compared to AlClPc (2.6 h) and AlPc–PEG (23 min), lower retention by liver and spleen and higher tumour-to-skin and tumour-to-muscle ratios. Our data demonstrate that addition of hydrophilic axial ligands to AlPc, while modifying in vitro and in vivo kinetics, does not reduce the PDT efficiency of the parent molecule. Moreover, in the case of the polyvinylalcohol derivative, axial coordination confers advantageous pharmacokinetics to AlPc, which makes this photosensitizer a valuable, water soluble candidate drug for clinical PDT of cancer.

**Keywords:** Colo-26 tumour; EMT-6 tumour; aluminium phthalocyanine; photodynamic therapy; polyethyleneglycol; polyvinylalcohol

Among the second-generation photosensitizers (PS) developed for the treatment of neoplastic diseases by photodynamic therapy (PDT) (Kessel, 1996; Ochsner, 1997), metallo-phthalocyanines (MePc) have been proposed (Spikes, 1986; van Lier, 1990; Rosenthal, 1991) as an alternative to Photofrin (PII), the only PS currently approved for clinical use. Their stronger absorption in the red part of the spectrum (molar extinction coefficient, $2.5 \times 10^5$ $M^{-1}$ $cm^{-1}$ at 675 nm), where the depth of light penetration in tissues is twice that obtained at 630 nm with PII (Svaasand, 1984), together with their chemical homogeneity and their lower potential to induce cutaneous photosensitivity (Roberts et al, 1989; Tralau et al, 1989) are major advantages over PII.

Unsubstituted MePc are not soluble in physiological solvents and their in vivo administration relies upon their incorporation into carriers, such as the liposomal formulation of ZnPc (Isele et al, 1995), or their chemical conversion into water-soluble dyes by the attachment of selected substituents onto the benzene rings of the macrocycle. An inverse relationship was related between the degree of substitution by sulphonato groups of MePc and their hydrophobicity and photodynamic activities, both at the cellular level and in tumour-bearing mice (Brasseur et al, 1987, 1988; Paquette et al, 1988; Berg et al, 1989; Chan et al, 1990, 1991; Margaron et al, 1996*a*). The potential of the water-soluble aluminium sulphophthalocyanine (AlPcS) to generate activated oxygen species (Sharman et al, 1999) and to induce a photodynamic response in vivo has been widely evaluated, and a mixture of AlPcS bearing 2–4 sulphonato groups per Pc (Photosense) is used extensively and successfully in clinical PDT in Russia (Zharkova et al, 1994). Furthermore, the di-sulphonated derivative has been shown to induce tumour regression mainly via direct tumour cell kill rather than damage to the tumour vasculature, as observed for PII (Chan et al, 1996; Margaron et al, 1996*b*). The unsubstituted AlClPc has, however, attracted less attention even though it has been shown that this compound formulated as a Cremophor emulsion was preferentially retained by a gliosarcoma and was able to induce tumour necrosis in this model (Dereski et al, 1994). More recently, we demonstrated that the Cremophor-formulated AlClPc was more effective in inducing tumour regression in EMT-6 tumour-bearing mice than the mono- through tetrasulphonated derivatives, while exerting relatively minor effects against normal tissues (Chan et al, 1997). The low dosage required together with the absence of systemic toxicity, even at much higher doses, renders the AlClPc Cremophor emulsion one of the most potent photosensitizer preparations currently available in terms of therapeutic window. However, Cremophor oil is known to induce unwanted side-effects in patients (Dye and Watkins,

*Received 13 October 1998*
*Revised 1 February 1999*
*Accepted 11 February 1999*

*Correspondence to:*: JE van Lier

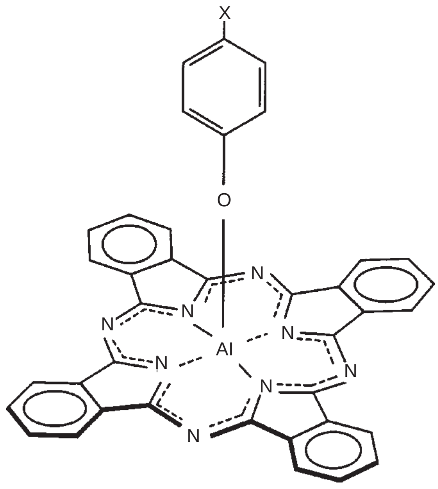

**Figure 1** Structural formula of AlPc–PEG (**5**) (x = -O-PEG) and AlPc-PVA (**10**) (x = $-(CH_2)_2CONH(CH_2)_3NH$-PVA). See also Schemes 1 and 2

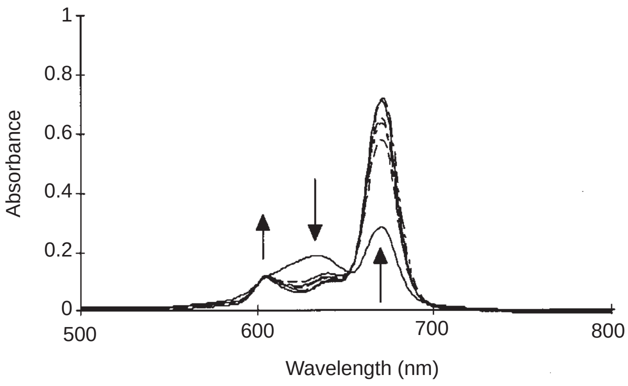

**Figure 2** UV-vis spectrum of AlPc–PEG at 1 min, 30 min, 45 min, 1 h, 16 h and 24 h after dilution at 2.8 $10^{-6}$ M in DMF. The peaks characteristic for the monomeric dye at 604 and 670 nm increase while the peak of the aggregate at 634 nm decreases

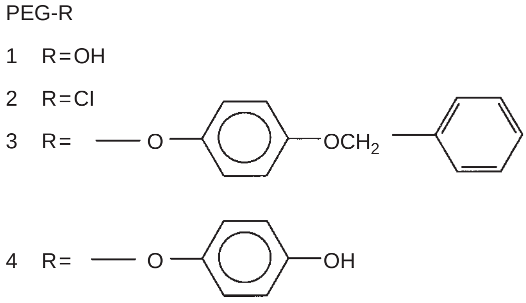

Scheme 1

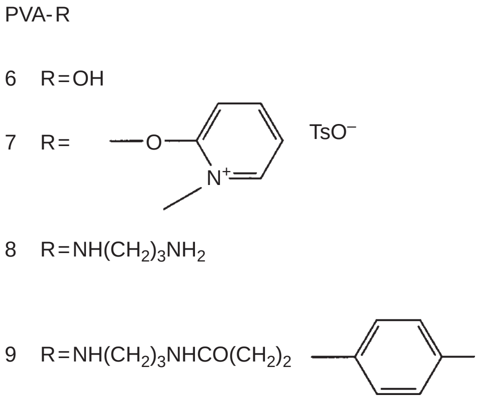

Scheme 2

1980). On such account we conjectured that the coordination of a biologically acceptable polymer on the central $Al^{3+}$ ion would confer water solubility to AlPc without changing the electronic structure and photochemical properties, and without adding any isomerism. By adapting the reaction of Koenigs–Knorr, it was possible to attach a phenolic polyethyleneglycol (PEG) or a phenolic polyvinylalcohol (PVA) via a coordination bond, replacing the Cl substituent of AlClPc, to obtain water-soluble AlPc–PEG and –PVA conjugates. The photodynamic activity of these two analogues was evaluated and compared with that of the unsubstituted AlClPc in vitro against the EMT-6 mouse mammary tumour cell line and in vivo against the EMT-6 and the colon carcinoma Colo-26 tumours implanted intradermally (i.d.) in Balb/c mice. Pharmacokinetics were performed in the EMT-6 tumour-bearing mice. In spite of differences in the biodistribution pattern of the three dye preparations, the coordination of polymeric, axial ligands to AlPc does not reduce its photoefficiency and results in potent, water-soluble photosensitizer preparations.

### MATERIALS AND METHODS

### Chemistry

#### *General*

The UV-vis spectra were recorded with a Hitachi U-2000 spectrophotometer. All solvents and chemicals were purchased from Aldrich Chemical Co. (Milwaukee, WI, USA) and were used without further purification. PEG with a MW 2000 g and an average degree of polymerization of 43, PVA (MW 13–23 × $10^3$) and AlClPc, were obtained from Aldrich. The Bolton-Hunter reagent was obtained from Pierce (Rockford, IL, USA).

#### *Reaction of AlClPC with PEG-2000*

In order to activate the PEG (**1**) to react with AlClPc, the material was converted in a three-step reaction to a phenoxy derivative (**4**) (Scheme 1). Briefly this involved substitution of the free hydroxyl groups with chloro groups through a reaction with thionyl chloride to yield **2**, followed by a reaction with 4-benzyloxyphenol to yield the protected benzylphenoxy-PEG derivative (**3**) (Casnati et al, 1997) which upon hydrolysis gives the activated phenoxy-PEG (**4**). Reaction of **4** with AlClPc, silver carbonate and silver perchlorate under Koenigs–Knorr conditions in DMF (Egan, 1972; Wulff and Röhle, 1974) afforded the axial substituted AlPc–PEG (**5**) (Figure 1). The mass spectra of all PEG derivatives failed to show any molecular ions above 2500. Evidence of the reaction with AlClPc was ascertained by the water solubility of the axial coordinated AlPc–PEG (**5**) and its characteristic UV-vis spectrum (Figure 2). The peak at 670 nm, typical for monomeric

 *© 1999 Cancer Research Campaign*

AlPc, increases with time after dilution of **5** in DMF, suggesting aggregation of AlPc–PEG in aqueous solution. Direct mixing of AlClPc and PEG did not give a blue aqueous solution, confirming that PEG alone was not able to solubilize AlClPc.

### *Reaction of AlClPc with PVA*

The PVA was activated as a phenoxy derivative attached to the PVA via an amine substituent (Scheme 2). Briefly, the PVA (**6**) was reacted with 2-fluoro-*N*-methylpyridinium p-toluene-sulphonate to activate the hydroxyl groups, i.e. compound **7** (Jiang et al, 1990), that were then attacked by the nucleophilic 1,3-diaminopropane to give the polyamino-PVA **8**. An average of ten amines per PVA was calculated after the reaction with sodium benzenetrinitrosulphonate (Fields, 1972). Compound **8** was then coupled via an amide linkage using the Bolton-Hunter reagent, to yield the activated phenolic PVA (**9**). This phenolic polymer was condensed with AlClPc as described for PEG above to give the axial substituted AlPc–PVA **10** (Figure 1), which was characterized by its UV-vis absorption spectrum. Details of the synthetic procedures will be published elsewhere.

The final stock solutions of the conjugates remained stable, even after 3 months at room temperature, as evidenced by the absence of precipitate (0.45 μm-filter) and integrity of the absorption spectrum.

### Biological studies

### *Drug formulation*

AlClPc was formulated in an oil emulsion containing 10% Cremophor EL (Sigma) and 3% 1,2-propanediol (Sigma) in phosphate-buffered saline (PBS). After dissolution of AlClPc in a minimal volume of ethanol, Cremophor and 1,2-propanediol were added under sonication, whereafter ethanol was evaporated under vacuum. The solution was diluted with PBS pH 7.4 and filtered (Millipore, 0.22 μm). AlPc–PEG and AlPc–PVA were prepared in PBS. The final concentrations of the polymeric Pcs were determined spectroscopically 24 h after dilution in DMF to ensure complete monomerization ($\varepsilon = 2.5 \times 10^5$ $M^{-1}$ $cm^{-1}$ at ~670 nm). Solutions were kept in the dark and stored at room temperature.

### *Cells*

Mouse mammary tumour, EMT-6 cells were maintained in Waymouth's medium (Gibco, Burlington, Canada) supplemented with 15% fetal bovine serum (FBS, ICN, Aurora, Ohio) and 1% L-glutamin (Gibco). Mouse colon adenocarcinoma, Colo-26 cells (Tsuruo et al, 1983) were obtained from Dr B Henderson (Rosswell Park Cancer Institute, Buffalo, NY, USA) and maintained in vivo by successive intradermal inoculation of thawed cells and freezing of fresh cells prepared from homogenized tumours.

### *Cellular photoinactivation*

Cell survival was estimated by means of the colourimetric MTT (3-(4-5-dimethylthiazol-2-yl)-2,5-diphenyltetrazolium bromide) assay (Tada et al, 1986). Briefly, $15 \times 10^3$ EMT-6 cells per well were inoculated in 100 μl Waymouth's growth medium in 96-multi-well plates and incubated overnight at 37°C and 5% carbon dioxide. The cells were rinsed twice with PBS and incubated with 100 μl of the drug at 1 μM in Waymouth's medium containing 1% FBS for 1 or 24 h at 37°C and 5% carbon dioxide. After incubation, the cells were rinsed twice with PBS, refed with 100 μl Waymouth's medium and exposed to red light. The light source consisted of two 500 W tungsten/halogen lamps (GTE Sylvania, Montreal, Canada) fitted with a circulating, refrigerated, aqueous Rhodamine filter. The fluence rate calculated over the absorbance peak of the aluminium phthalocyanine (660–700 nm) was 10 mW $cm^{-2}$ for a maximum total fluence of 36 J $cm^{-2}$. The cells were incubated at 37°C and 5% carbon dioxide overnight before assessing cell viability. Fifty-microlitres of a fivefold diluted MTT stock solution (Aldrich, Milwaukee, WI, USA; 5 mg $ml^{-1}$ PBS) in Waymouth's medium were added to each well. After 3 h, 100 μl sodium dodecyl sulphate (SDS; Gibco) 10% in 0.01 N hydrochloric acid was added in the wells. Plates were incubated overnight at 37°C whereafter the absorbance was read at 595 nm by means of a microplate reader (Bio-Rad, Mississauga, Ontario, Canada). The average absorbance of the blank wells in which cells were omitted was subtracted from the readings of the other wells. The average absorbance of the control cells, which were incubated with dye-free Waymouth's 1% FBS, represents 100% cell survival. The light dose required to inactivate 90% of the cells ($LD_{90}$) was extrapolated from the survival curves. Eightfold replicates were run per drug and light dose and each experiment was repeated at least three times.

### *Tumour models*

Experiments were performed on male BALB/c mice (18–22 g) (Charles River Breeding Laboratories, Montreal, Canada) bearing the EMT-6 or the Colo-26 tumour following a protocol approved by the Canadian Council on Animal Care and an in-house ethics committee. The animals were allowed free access to water and food throughout the course of the experiments. Before tumour implantation, hair on the hind legs and back of the mice was removed by shaving and chemical depilating (Nair, Whitehall, Mississauga, Canada). One EMT-6 tumour (2–3 for the biodistribution) was implanted on each hind thigh by i.d. injection of $2 \times 10^5$ cells suspended in 0.05 ml growth medium. In the case of the Colo-26 tumour, one tumour was implanted i.d. on the right hind thigh only, by inoculation of $1 \times 10^6$ Colo-26 cells, to allow for a longer follow-up of the tumour regrowth after PDT.

### *Biodistribution*

Mice were used 10 or 11 days after cell inoculation when the tumour diameter and thickness reached 4–8 mm and 2–4 mm respectively. Tumour-bearing mice were injected intravenously (i.v.) via the caudal vein with 2 μmol $kg^{-1}$ of dye (0.2 ml per 20 g body weight). At different time intervals after dye administration (5 min to 1 week), blood was collected by intracardiac puncture by means of a heparinized syringe, whereafter the animals ($n = 4$ per time interval) were sacrificed. The blood was centrifuged in Eppendorf tubes for 5 min at 2000 ***g*** and the plasma was collected. One hundred microlitres of plasma or blood were mixed with 1.9 ml DMF. Organs and tissues of interest were removed, washed with saline (0.9%) and blotted dry. Whole tumours (2–3), aliquots of other organs and minced skin (80–150 mg) were homogenized with a 20-fold volume of DMF using a Polytron® fitted with a PT 10/35 rotor (Brinkmann, Mississauga, Canada). After overnight incubation at room temperature, the homogenates were centrifuged at 4°C (3500 rpm for 20 min). The dye concentration in the clear supernatant was assayed by fluorescence (Fluorescence spectrophotometer F-2000, Hitachi, Tokyo, Japan)

© 1999 Cancer Research Campaign 

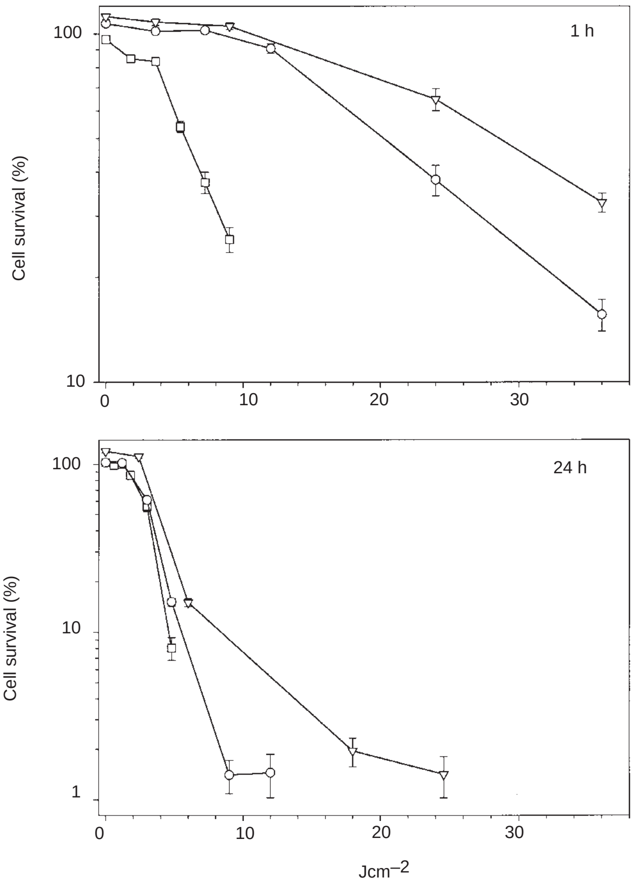

**Figure 3** Effect of light dose (J $cm^{-2}$) on EMT-6 cell survival after 1 h (top) and 24 h (bottom) incubation with 1 μM AlClPc (□), AlPc–PEG (○) and AlPc–PVA (∇)

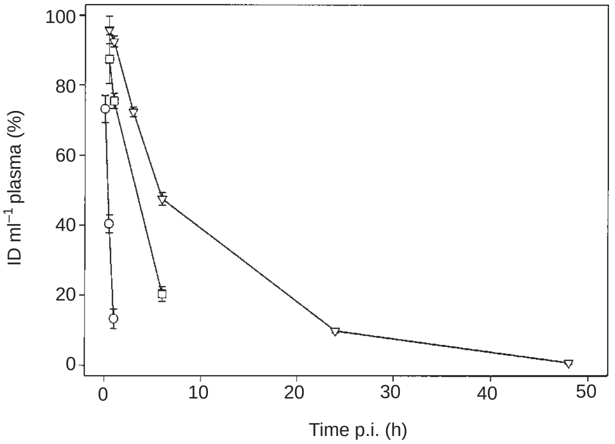

**Figure 4** Plasma clearance (mean ± sem) of AlClPc (□), AlPc-PEG (○) and AlPc–PVA (∇) after iv injection of 2 μmol $kg^{-1}$ in EMT-6 tumour bearing mice ($n$ = 4)

($\lambda_{ex}$ 606 nm, $\lambda_{em}$ ~680 nm; band pass 5 nm and 10 nm respectively). Calibration curves were established by adding known amounts of dye to 100 μl plasma or blood or 80–150 mg of tissue samples from control mice, whereafter the tissues were treated as described above. No fluorescence was found in control tissue samples to which no dye had been added. Plasma half-lives were calculated by means of the Pharmkit program (version 3.00; Johnston and Woollard R, 1990).

### *Photodynamic therapy*

For PDT studies, mice were used 6–8 days after tumour inoculation (tumour size: 3–5 mm diameter, 2–3 mm thickness). Animals were given an i.v. injection of drugs at 0.25–10 μmol $kg^{-1}$ (0.2 ml per 20 g body weight) and the right tumour was treated with red light 24 h later. In the case of the EMT-6 tumour, the left tumour served as a control and animals were discarded from the analysis when the control tumour underwent spontaneous regression or showed abnormally slow growth. This was never observed with Colo-26 tumour model in control animals. Tumours were illuminated with a 8 mm diameter beam of 650–700 nm light (200 mW $cm^{-2}$ for a total fluence of 400 J $cm^{-2}$) generated by a 1000 W Xenon lamp, equipped with a 10 cm circulating water filter and two glass filters (Corion LL650 and LS700, Holliston, MA, USA). A positive tumour response was assigned to tumours that appeared macroscopically as flat and necrotic tissues within a few days after PDT. Animals were considered cured when a complete tumour regression, defined as the absence of a palpable tumour, was seen at 3 weeks after PDT. In the case of the Colo-26 tumour, the percentage of cured animals was low. Therefore, the anti-tumour activity was further evaluated in non-cured mice through a growth-delay analysis. Tumour volume was measured externally with a calliper according to an hemiellipsoid model [$V = 1/2\ (4\pi/3) \times (l/2) \times (w/2) \times h$, l = length, w = width, h = height]. No correction was made for skin thickness. Tumour regrowth curves were plotted for each treated tumour and the time in days for a tumour to grow to 20 times its initial volume at the time of phototreatment (Vo) was determined. Then, the mean (± s.e.m.) was calculated for each treatment group ($n$ = 6–8). In the case of the controls, the volume of 45 untreated tumours was plotted against the time post-inoculation and the time in days required to reach 20 times the mean initial volume of the treated tumours group at the time of phototreatment (Vo: 8.9 ± 0.6 $mm^3$) was determined from the regression curve (Figure 6A). The significance of the difference in tumour growth time between the treated and the control group and among the treated groups was assessed by the Student's $t$-test for unpaired values.

### RESULTS

### Cellular photoinactivation

Photosensitization of EMT-6 cells by AlPc derivatives is shown in Figure 3 after 1 and 24 h incubation with 1 μM dye. No dark cytotoxicity was observed under these conditions. The light dose required to kill 90% of cells ($LD_{90}$) after 1 h incubation was 13.5 J $cm^{-2}$ for AlClPc and more than 36 J $cm^{-2}$ for AlPc–PEG and AlPc–PVA. Increasing the incubation times to 24 h decreased the $LD_{90}$ to 4.5, 6 and 8.5 J $cm^{-2}$ respectively.

### Biodistribution studies

Plasma clearance of AlClPc and AlPc conjugates **5** and **10** is shown in Figure 4. The half-life was 23 min, 2.6 h and 6.8 h for AlPc–PEG (**5**), AlClPc and AlPc–PVA (**10**) respectively. A high

 © 1999 Cancer Research Campaign

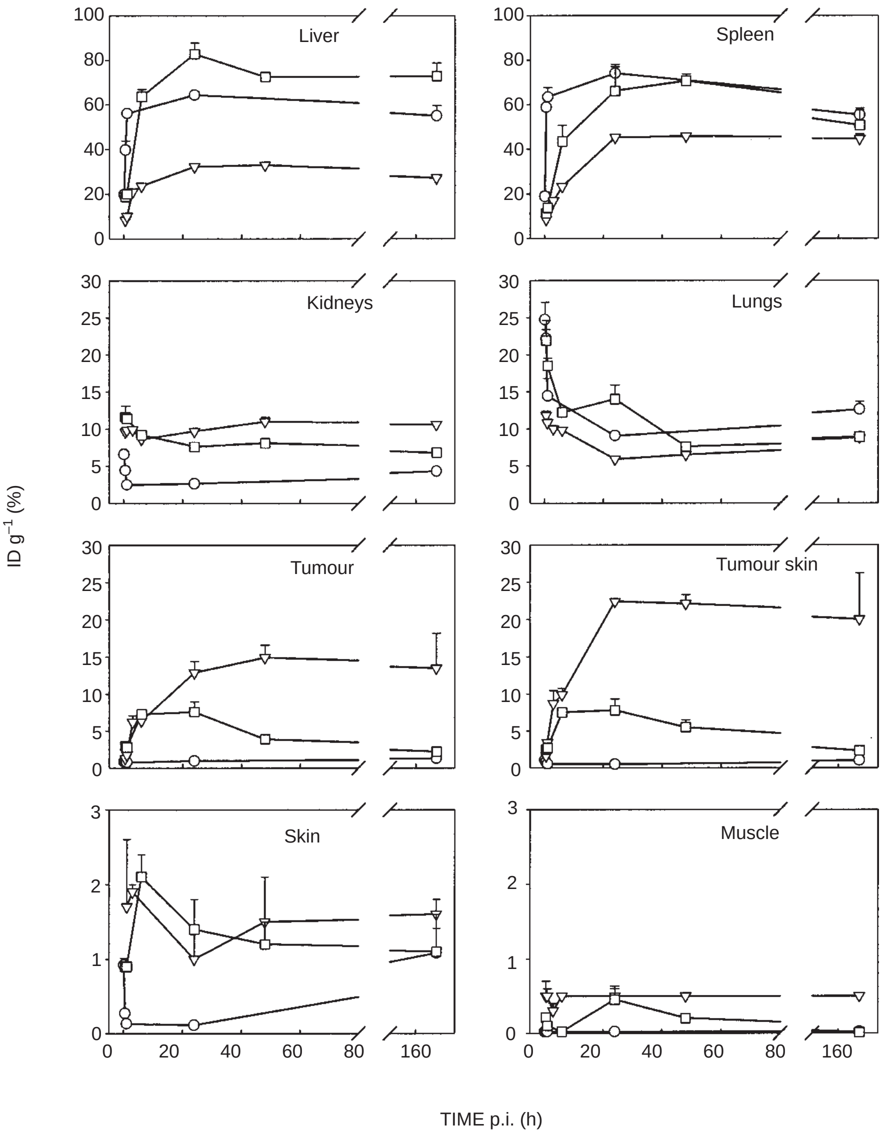

**Figure 5** Tissue concentrations in %ID g$^{-1}$ (mean ± s.e.m.) of AlClPc (□), AlPc-PEG (○) and AlPc-PVA (∇) as a function of time after i.v. injection of 2 μmol kg$^{-1}$ in EMT-6 tumour bearing mice ($n$ = 4)

**Table 1** Tumour-to-plasma and tumour-to-tissues ratios for AlClPc, AlPc–PEG and AlPc–PVA as a function of time (h) after i.v. injection of 2 μmol kg$^{-1}$ in EMT-6 tumour-bearing mice ($n$ = 4)

| | 6 h | 24 h | 48 h | 168 h |
|---|---|---|---|---|
| *AlClPc in Cremophor emulsion* | | | | |
| Tumour-to-plasma | 0.4 | >> | >> | >> |
| Tumour-to-skin | 3.5 | 5.3 | 3.3 | 2.0 |
| Tumour-to-tumour skin | 1.0 | 1.0 | 0.7 | 0.9 |
| Tumour-to-muscle | – | 16.9 | 19.5 | >> |
| *AlPc-PEG in PBS* | | | | |
| Tumour-to-plasma | – | >> | – | >> |
| Tumour-to-skin | – | 8.8 | – | 1.2 |
| Tumour-to-tumour skin | – | 1.9 | – | 1.3 |
| Tumour-to-muscle | – | >> | – | >> |
| *AlPc-PVA in PBS* | | | | |
| Tumour-to-plasma | 0.1 | 1.3 | 21.3 | >> |
| Tumour-to-skin | – | 12.5 | 9.8 | 8.2 |
| Tumour-to-tumour skin | 0.6 | 0.6 | 0.7 | 0.7 |
| Tumour-to-muscle | 12.6 | 26.9 | 32.3 | 27.0 |

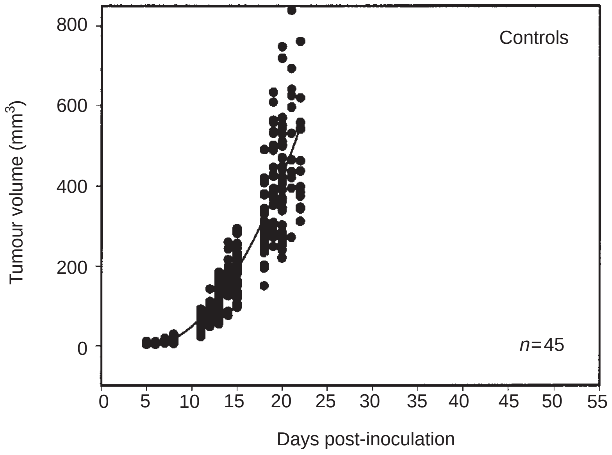

**Figure 6** Growth of Colo-26 tumour in control mice ($n$ = 45)

**Table 2** Tumour response induced in Balb/c mice by AlPc- and PII-mediated PDT (24 h p.i., 200 m W $cm^{-2}$, 400 J $cm^{-2}$)

| PDT treatment | Drug dose µmol $kg^{-1}$ ($mg\ kg^{-1}$) | *n* | Vo[a] $mm^3$ (sem) | Tumour response (% of mice) Regression[b] | Cure[c] | Oedema[d] | Regrowth to 20 Vo[e] days (s.e.m.) |
|---|---|---|---|---|---|---|---|
| EMT-6 tumour | | | | | | | |
| AlClPc[f] | 0.25 | 8 | 8.7 (0.7) | 100 | 100 | + | NA |
| AlPc–PEG | 0.25 | 8 | 14.9 (1.1) | 100 | 75 | ++ | NA |
| AlPc–PVA | 0.25 | 8 | 11.9 (0.9) | 100 | 100 | +++ | NA |
| PII[g] | (5) | 10 | NA | 70 | 70 | +++ | NA |
| Colo-26 tumour | | | | | | | |
| Controls | – | 45 | 8.9 (0.6)[h] | – | – | – | 15.0 (0.3) |
| AlClPc | 2 | 10 | 10.3 (1.2) | 80 | 30 | ++ | 23.5 (2.1) |
| AlPc–PEG | 2 | 9 | 8.3 (0.5) | 100 | 30 | +++[i] | 30.1 (1.7) |
| AlPc–PVA | 2 | 9 | 10.3 (1.4) | 90 | 30 | +++ | 32.4 (3.4) |
| PII | (10) | 8 | 7.9 (0.7) | 100 | 0 | +++ | 24.0 (0.8) |

[a]Tumour volume at the time of PDT. [b]Flat and necrotic within 3–5 days after PDT. [c]Complete tumour regression at 14 days (EMT-6, PII) or 21 days (EMT-6, Pc) or 30 days minimum (Colo-26) post-inoculation. [d]Observed 24 h after PDT, (+) light oedema, (++) oedema, (+++) extensive oedema. [e]Time required for a tumour to reach 20 times the volume at the time of PDT, cured mice were excluded from this analysis. [f]From Chan et al (1997). [g]From Brasseur et al (1996). [h]Mean tumour volume of the 4 treated groups of non cured tumours (*n* = 27) was used to calculate 20 Vo in the control group (*n* = 45). [i]Damage to surrounding muscle, limiting leg mobility and lasting for at least 35 days post-PDT was observed in 30% of mice.

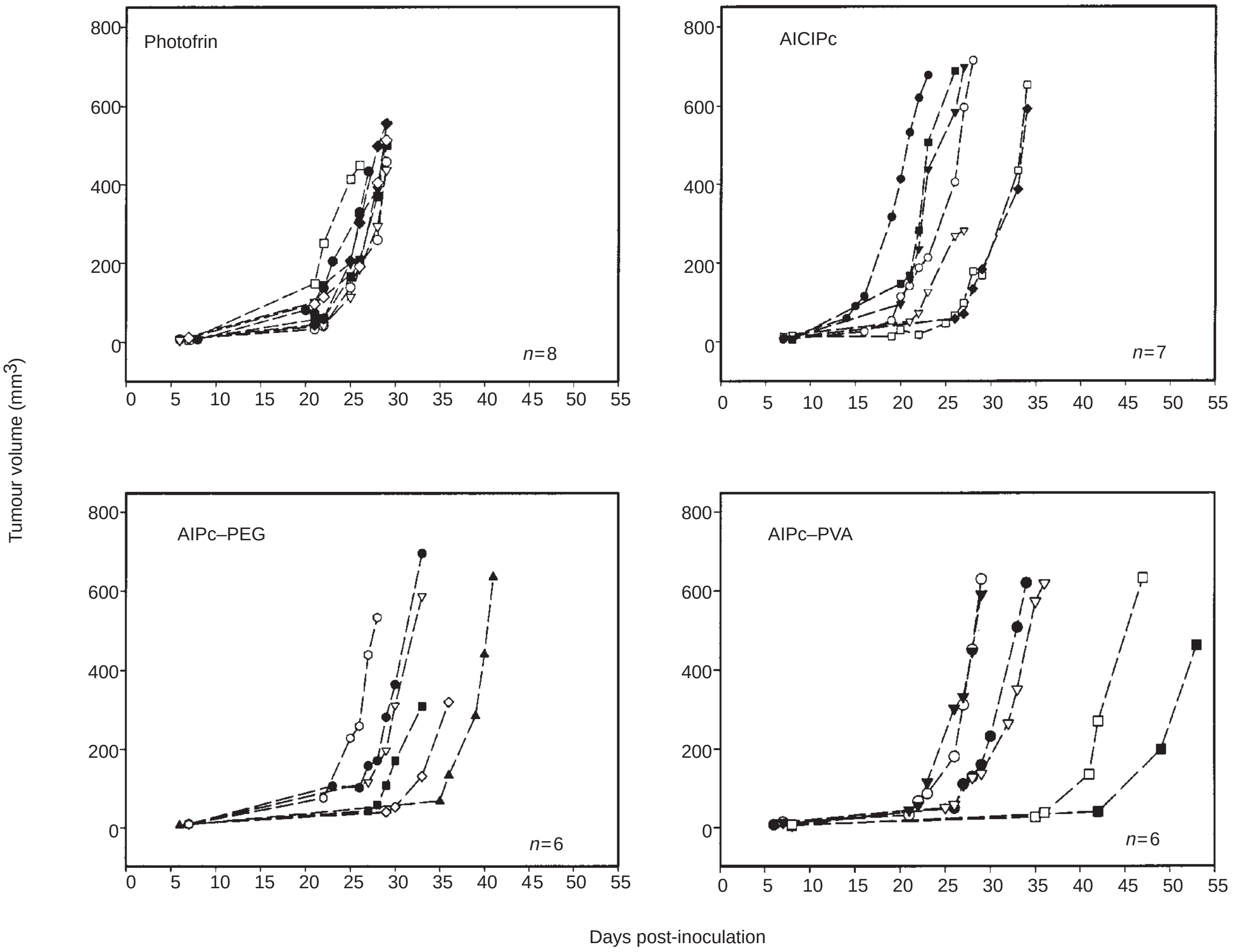

**Figure 7** Growth of Colo-26 tumour in mice treated with AlClPc, AlPc-PEG and AlPc-PVA at 2 µmol $kg^{-1}$ and Photofrin at 10 mg $kg^{-1}$ following PDT at day 6 or 7 after tumour inoculation (*n* = 6–8). Cured mice were excluded from this analysis

© 1999 Cancer Research Campaign retention of the dye by the organs of the reticulo-endothelial system, i.e. liver and spleen, was seen with all three preparations but was much more elevated for AlClPc and AlPc–PEG (Figure 5). Uptake by the lungs was also higher while elimination by the kidneys was somewhat lower for AlClPc and AlPc–PEG as compared to AlPc–PVA. Tumour uptake of AlClPc increased gradually from 30 min to 6–24 h post-injection (pi), whereafter it decreased. In the case of AlPc–PVA, the tumour uptake was higher at 24 h pi and remained stable for 1-week pi. Tumour and tumour-skin retentions of AlPc–PEG were much lower as compared to AlClPc and AlPc–PVA. For these two dyes respectively, the skin overlying the tumour accumulated equivalent or higher amount of dye than the skin removed at sites away from the tumour (leg). This correlates with high vascularization of the skin surrounding the tumour. Retention of AlPc by normal skin and muscle was low for all preparations. Tumour-to-tissue ratios are presented in Table 1. The highest tumour-to-skin and tumour-to-muscle ratios were obtained for AlPc–PVA.

### Photodynamic therapy

Table 2 presents the tumour response induced by AlPc-mediated PDT in the EMT-6 and Colo-26 tumour models implanted in Balb/c mice. Complete EMT-6 tumour regression was achieved in 75% of animals or more with 0.25 μmol $kg^{-1}$ for all three dye preparations. For comparison, similar tumour control was obtained with 5 mg $kg^{-1}$ Photofrin. In the Colo-26 tumour model, complete tumour regression was found in 30% of mice with 2 μmol $kg^{-1}$ for all three dyes. However, differences were noted in the tumour growth delay (t in days) measured in the non-cured animals (Figure 6 and 7, Table 2). Significantly longer delays were obtained for all three dye preparations as compared to the control tumours ($P < 0.01$). In addition, AlPc–PVA ($t = 32.4 \pm 3.4$) and AlPc-PEG ($t = 30.1 \pm 1.7$) gave rise to better growth delay as compared to AlClPc ($t = 23.5 \pm 2.1$, $P = 0.04$). In the case of Photofrin, no tumour cure was observed with 10 mg $kg^{-1}$ while the tumour growth delay extended to $24 \pm 0.8$ days, which was different from the control ($t = 15 \pm 0.3$, $P < 0.01$) and from AlPc–PVA ($P = 0.02$) and AlPc–PEG ($P < 0.01$). Oedema was always less important with AlClPc than with PII and the two polymeric AlPc derivatives. Impaired mobility of the treated leg reflecting persistent damage to muscle was seen in 30% of animals, treated with AlPc–PEG at 2 μmol $kg^{-1}$. In contrast, AlClPc and AlPc–PVA at the same dose did not induce damage to leg muscle.

### DISCUSSION

Various photosensitizers have been conjugated to polymeric carriers including chlorine to *N*-(2-hydroxypropyl)methacrylamide (Krinick et al, 1994) or to cationic, anionic or neutral poly-L-lysine (Soukos et al, 1997). Substitution of poly-L-lysine chlorine conjugates with PEG increases phototoxicity towards cancer cells (Hamblin et al, 1998; Rizvi and Hasan, 1998). A tetra-substituted PEG conjugate of the meta-(tetrahydroxyphenyl)-chlorine (m-THPC, Foscan, Temoporfin) is under extensive study and shows extended plasma half-life, tumour selectivity (Morlet et al, 1997; Rheinwald et al, 1998; Rovers et al, 1998) and good PDT efficacy (Krüger et al, 1998). Three to four methoxy-PEG (MW 5000) linked to tetra-(4-hydroxyphenyl)-porphin (Pomer et al, 1995) improved tumour accumulation in human renal cell carcinoma transplanted in nude mice as compared to dihaemato-porphyrin ether (DHE). In contrast, a water-soluble silicon-naphthalocyanine (SiNc) obtained by coordination of two PEG ligands (MW 1900) in axial positions to the central metal ion (Bellemo et al, 1992) showed poor tumour selectivity and no phototherapeutic activity in a MS-2 fibrosarcoma transplanted in Balb/c mice, while hydrophobic derivatives of SiNc delivered in liposomes (Cuomo et al, 1990) or in Cremophor emulsion (Brasseur et al, 1994) are good tumour photosensitizers. Modified PVA (MW 10 000) was also loaded with benzoporphyrin derivative (M-PVA-BPD) and tested at a molar ratio of 1:25 in the rhabdomyosarcoma implanted in DBA/2 mice (Davis et al, 1993). Although the level of M-PVA-BPD in tumour tissue was lower than that of free BPD, it was shown by an in vivo/in vitro assay that the conjugate was bound more tightly to the tumour cells and effected higher tumour cell kill when activated by light.

We now described the conjugation of PEG and PVA to AlClPc via an axial coordination bond. The photodynamic potential of the polymeric AlPc conjugates was first evaluated at the cellular level. The AlClPc emulsion was more phototoxic than AlPc–PEG and AlPc–PVA after 1 h incubation, reflecting its faster rate of cellular uptake. However, after 24 h incubation, differences in photoactivity among the three dye preparations were much less pronounced. AlClPc photoactivity is consistent with literature data (Ben-Hur and Rosenthal, 1986; Chan et al, 1987; Daziano et al, 1996; Rasch et al, 1996). However, among the phthalocyanines evaluated in our laboratory, these AlPc derivatives are not the most active on cell cultures. The monosulphododecafluoro zinc Pc derivative, $ZnPcF_{12}S_1$, for example, was much more active ($LD_{90}$ 24 h inc.: 0.25 J $cm^{-2}$) but this substantial cellular photoinactivation correlated with high level of mortality after PDT in mice (Allémann et al, 1997). In contrast, in terms of drug dosage required for efficient in vivo PDT, AlClPc and its two polymeric conjugates are among the most active phthalocyanines tested to date in our laboratory (Larroque et al, 1996; Chan et al, 1997; Hu et al, 1998). This again confirms that the level of in vitro photoinactivation is not predictive for in vivo photoefficacy.

Biodistribution studies performed in EMT-6 tumour-bearing mice revealed important differences among the three dyes in the rate of plasmatic clearance as well as in the level of tissue uptake. It is evident that the delivery vehicle and the polymeric moiety affects the tissue distribution of AlPc substantially. It is well known that Cremophor increases the plasma circulation time of a drug by altering plasma lipoproteins and promotes its distribution to neoplastic tissues (Woodburn et al, 1994), as also observed for the Cremophor emulsion of AlClPc ($t_{1/2}$ 2.6 h). However, important and persistent retention of AlClPc by the liver (73% at 1-week pi) could result in hepatic toxicity. In contrast, AlPc-PEG was cleared readily from the circulation ($t_{1/2}$ 23 min), while the dye retention by the reticulo-endothelial system was rapid and elevated resulting in a very low tumour uptake. These data are in agreement with a study on the effect of the molecular weight (MW) of the PEG on its half-life in circulation and its body distribution (Yamaoka et al, 1994). Small PEG polymers, such as used in our study (MW 2000), tend to freely translocate from the circulation to extravascular tissues, whereas large PEGs translocate more slowly. The plasma half-life increases from 18 min to 1 day with increasing PEG MW from 6000 to 190 000, whereas liver clearance increases with the increasing PEG MW. The most interesting pharmacokinetic profile was obtained with AlPc–PVA, which showed the longest plasma circulation time ($t_{1/2}$ 6.8 h). This effect on the rate of plasmatic clearance could relate to the higher MW of the polymer

(13 000–23 000), when compared to AlPc–PEG as well as to its increased hydrophilicity when compared to AlClPc. AlPc–PVA also exhibited the lowest retention by the liver, spleen and lungs, the best tumour-to-skin and tumour-to-muscle ratios (12 and 27 respectively at 24 h pi) as well as the highest and most persistent tumour uptake, which allows for repeated PDT treatments.

PDT of the EMT-6 tumour showed that water-soluble AlPc conjugates were able to induce substantial tumour control at the same low drug dose (0.25 μmol $kg^{-1}$) as unsubstituted AlClPc. Due to the well-known immunogenicity of the EMT-6 tumour (Henderson et al, 1985), the photodynamic potential of the conjugates was further evaluated on the Colo-26 tumour model. The absence of immunogenicity of the latter tumour may explain, at least in part, the lower level of tumour regression (30% of mice) obtained at higher drug dose (2 μmol $kg^{-1}$). However, significantly longer tumour regrowth delays were observed in the non-cured treated mice as compared to untreated animals. A two-time increase was observed in the case of AlPc–PVA and AlPc–PEG, while in the case of AlClPc and PII (10 mg $kg^{-1}$) the growth delay was only 1.5 times that of the controls. Surprisingly, despite very low tumour uptake as compared to AlClPc and AlPc–PVA, AlPc–PEG was able to induce a similar tumour control rate. Also, plasma levels of AlPc–PEG at the time of phototreatment were below the detection limit. This suggests that the residual amount of dye present in the tumour is distributed to sites of higher photodynamic sensitivity. While the photodynamic potential of AlClPc was maintained or even increased by addition of a PEG or PVA molecule, the low extent of oedema induced with the unsubstituted AlClPc was lost. This does not seem to be related to differential dye retention by the muscle or skin, since the uptake by these tissues is very similar and quite low for all three dye preparations. However, more important damages to the muscle surrounding the tumour were observed with AlPc–PEG as compared to AlPc–PVA.

In conclusion, we have shown that the water-soluble AlPc–PVA and AlPc–PEG conjugates exhibit photodynamic activities similar or superior to that of the unsubstituted AlClPc. The relatively low drug dosage required to induce tumour control in the two different models suggests that these conjugates are among the most active water-soluble photosensitizers currently under evaluation. Finally, AlPc–PVA has the advantage of lower splenic and hepatic retentions and less incidence of damage to normal tissues as compared to AlPc–PEG.

### ACKNOWLEDGEMENTS

This research was supported by the Medical Research Council of Canada. JEvL is the holder of the Jeanne and J-Louis Lévesque Chair in Radiobiology. The authors are grateful to Virginie Moucadel for technical assistance.

### REFERENCES

Allémann E, Brasseur N, Kudrevich SV, La Madeleine C and van Lier JE (1997) Photodynamic activities and biodistribution of fluorinated zinc phthalocyanine derivatives in the murine EMT-6 tumor model. *Int J Cancer* **72**: 289–294

Bellemo C, Jori G, Rihter BD, Kenney ME and Rodgers MAJ (1992) Si(IV)-naphthalocyanine: modulation of its pharmacokinetic properties through the use of hydrophilic axial ligands. *Cancer Lett* **65**: 145–150

Ben-Hur E and Rosenthal I (1986) Photosensitization of chinese hamster cells by water-soluble phthalocyanines. *Photochem Photobiol* **43**: 615–619

Berg K, Bommer JC and Moan J (1989) Evaluation of sulfonated aluminum phthalocyanines for use in photochemotherapy. A study on the relative efficiencies of photoinactivation. *Photochem Photobiol* **49**: 587–594

Brasseur N, Ali H, Langlois R and van Lier JE (1987) Biological activities of phthalocyanines – VII. Photoinactivation of V-79 chinese hamster cells by selectively sulfonated gallium phthalocyanines. *Photochem Photobiol* **46**: 739–744

Brasseur N, Ali H, Langlois R and van Lier JE (1988) Biological activities of phthalocyanines – IX. Photosensitization of V-79 chinese hamster cells and EMT-6 mouse mammary tumor by selectively sulfonated zinc phthalocyanines. *Photochem Photobiol* **47**: 705–711

Brasseur N, Nguyen T-L, Langlois R, Ouellet R, Marengo S, Houde D and van Lier JE (1994) Synthesis and photodynamic activities of silicon 2,3-naphthalocyanine derivatives. *J Med Chem* **37**: 415–420

Brasseur N, Lewis K, Rousseau J and van Lier JE (1996) Measurement of tumor vascular damage in mice with $^{99m}$Tc-MIBI following photodynamic therapy. *Photochem Photobiol* **64**: 702–706

Casnati A, Ferdani R, Pochini A and Angaro R (1997) p-(Benzyloxy) calix[8] arene: one-pot synthesis and functionalization. *J Org Chem* **62**: 6236–6239

Chan W-S, Marshall JF, Svensen R, Phillips D and Hart IR (1987) Photosensitising activity of phthalocyanine dyes screened against tissue culture cells. *Photochem Photobiol* **45**: 757–761

Chan W-S, Marshall JF, Svensen R, Bedwell J and Hart IR (1990) Effect of sulfonation on the cell and tissue distribution of the photosensitizer aluminum phthalocyanine. *Cancer Res* **50**: 4533–4538

Chan W-S, West CML, Moore JV and Hart IR (1991) Photocytotoxic efficacy of sulphonated species of aluminum phthalocyanine against cell monolayers, multicellular spheroids and in vivo tumours. *Br J Cancer* **64**: 827–832

Chan W-S, Brasseur N, La Madeleine C and van Lier JE (1996) Evidence for different mechanisms of EMT-6 tumor necrosis by photodynamic therapy with disulfonated aluminum phthalocyanine or photofrin: tumor cell survival and blood flow. *Anticancer Res* **16**: 1887–1892

Chan W-S, Brasseur N, La Madeleine C, Ouellet R and van Lier JE (1997) Efficacy and mechanism of aluminum phthalocyanine and its sulphonated derivatives mediated photodynamic therapy on murine tumours. *Eur J Cancer* **33**(11): 1855–1859

Cuomo V, Jori G, Rihter B, Kenney ME and Rodgers MAJ (1990) Liposome-delivered Si(IV)-naphthalocyanine as a photodynamic sensitizer for experimental tumors; pharmacokinetic and phototherapeutic studies. *Br J Cancer* **62**: 966–970

Davis N, Liu D, Jain AK, Jiang S-Y, Jiang F, Richter A and Levy JG (1993) Modified polyvinyl alcohol-benzoporphyrin derivative conjugates as phototoxic agents. *Photochem Photobiol* **57**: 641–647

Daziano JP, Steenken S, Chabannon C, Mannoni P, Chanon M and Julliard M (1996) Photophysical and redox properties of a series of phthalocyanines. Relation with their photodynamic activities on TF-1 and Daudi leukemic cells. *Photochem Photobiol* **64**: 712–719

Dereski MO, Madigan L and Chopp M (1994) Brain response to photodynamic therapy with Photofrin, monosulfonated aluminum phthalocyanine and tin purpurin. *Proc Spie* **2371**: 579–581

Dye D and Watkins J (1980) Suspected anaphylactic reaction to Cremophor EL. *Br Med J* **280**: 1353

Egan LP (1972) Synthesis and acid-catalyzed hydrolysis of 3-O-glycosyl-L-serine and -threonine. *Carbohydrate Res* **23**: 261–273

Fields R (1972) The rapid determination of amino groups with TNBS. *Methods Enzymol* **25**: 464–468

Hamblin MR, Miller JL, Rizvi I, Ortel B, Maytin EV and Hasan T (1998) Mechanism of differential phototoxicity after pegylation of poly-L-lysine chlorin E6 conjugates. *Proceedings of the 26th Annual Meeting of the American Society for Photobiology*, Snowbird, Utah, July 1998: 24S

Henderson BW, Waldow SM, Mang TS, Potter WR, Malone PB and Dougherty TJ (1985) Tumor destruction and kinetics of tumor cell death in two experimental mouse tumors following photodynamic therapy. *Cancer Res* **45**: 572–576

Hu M, Brasseur N, Yildiz SZ, van Lier JE and Leznoff CC (1998) Hydroxyphthalocyanines as potential photodynamic agents for cancer therapy. *J Med Chem* **41**: 1789–1802

Isele U, Schieweck K, Kessler R, Van Hoogevest P and Capraro HG (1995) Pharmacokinetics and body distribution of liposomal zinc phthalocyanine in tumour-bearing mice: influence of aggregation state, particle size, and composition. *J Pharm Sci* **84**: 166–173

Jiang FN, Jiang S, Liu D, Richter A and Levy JG (1990) Development of technology for linking photosensitizers to a model monoclonal antibody. *J Immunol Methods* **134**: 139–149

Kessel D (1996) Photodynamic therapy of neoplastic disease. *Drugs of Today* **32**: 385–396

Krinick NL, Sun Y, Joyner D, Spikes JD, Straight RC and Kopecek J (1994) A polymeric drug delivery system for the simultaneous delivery of drugs

 *© 1999 Cancer Research Campaign*

activatable by enzymes and/or light. *J Biomater Sci Polymer Edn* **5**: 303–324
Krüger TC, Mettler D, Schouwink H, Altermatt HJ and Ris HB (1998) Experimental assessment of photodynamic therapy with a polyethylene glycol derivative of mTHPC. *Proceedings of the 7th Biennial Congress of the International Photodynamic Association*, Nantes, France, July 1998: 66
Larroque C, Pelegrin A and van Lier JE (1996) Serum albumin as a vehicle for zinc phthalocyanine: photodynamic activities in solid tumour models. *Br J Cancer* **74**: 1886–1890
Margaron P, Grégoire M-J, Šcasnár V and Lier JE (1996*a*) Structure-photodynamic activity relationships of a series of 4-substituted zinc phthalocyanines. *Photochem Photobiol* **63**: 217–223
Margaron P, Madarnas P, Ouellet R and van Lier JE (1996*b*) Biological activities of phthalocyanines. XVII. Histopathologic evidence for different mechanisms of EMT-6 tumour necrosis induced by photodynamic therapy with disulfonated aluminum phthalocyanine or Photofrin. *Anticancer Res* **16**: 613–620
Morlet L, Vonarx V, Foultier MT, Gouyette A, Stewart C, Lenz P and Patrice T (1997) In vitro and in vivo spectrofluorometry of a water-soluble meta-(tetrahydroxyphenyl)chlorin (m-THPC) derivative. *J Photochem Photobiol B: Biol* **39**: 249–257
Ochsner M (1997) Photophysical and photobiological processes in the photodynamic therapy of tumours. *J Photochem Photobiol B: Biol* **39**: 1–18
Paquette B, Ali H, Langlois R and van Lier JE (1988) Biological activities of phthalocyanines – VIII. Cellular distribution in V-79 chinese hamster cells and phototoxicity of selectively sulfonated aluminum phthalocyanines. *Photochem Photobiol* **47**: 215–220
Pomer S, Grashev G, Sinn H, Kälble T and Staehler G (1995) Laser-induced fluorescence diagnosis and photodynamic therapy of human renal cell carcinoma. *Urol Int* **55**: 197–201
Rasch MH, Tijssen K, vanSteveninck J and Dubbelman TMAR (1996) Synergistic interaction of photodynamic treatment with the sensitizer aluminum phthalocyanine and hyperthermia on loss of clonogenicity of CHO cells. *Photochem Photobiol* **64**: 586–593
Rheinwald M, Wunder A, Sinn H, Haase T and Schlegel W (1998) Pharmacokinetic and photophysical properties of a polyethylene glycol derivative of mTHPC. *Proceedings of the 7th Biennial Congress of the International Photodynamic Association*, Nantes, France, July 1998: 67
Rizvi I and Hasan T (1998) Selective targeting of intraperitoneal tumors. *Proceedings of the 7th Biennial Congress of the International Photodynamic Association*, Nantes, France, July 1998: 107
Roberts WG, Smith KM, McCullough JL and Berns MW (1989) Skin photosensitivity and photodestruction of several potential photodynamic sensitizers. *Photochem Photobiol* **49**: 431–438
Rosenthal I (1991) Phthalocyanines as photodynamic sensitizers. Yearly review. *Photochem Photobiol* **53**: 859–870
Rovers J, Saarnak A, Sterenborg D, Terpstra O and Grahn M (1998) Biodistribution of mTHPC and its pegylated derivative SC 102 in a rat liver tumour model. *Proceedings of the 7th Biennial Congress of the International Photodynamic Association*, Nantes, France, July 1998: 67
Sharman WM, Allen CM and van Lier JE (1999) The role of activated oxygen species in photodynamic therapy. In: *Singlet Oxygen UV-A and Ozone, Methods in Enzymology*, Sies H and Packer L (eds). Academic Press: San Diego, CA
Soukos NS, Hamblin MR and Hasan T (1997) The effect of charge on cellular uptake and phototoxicity of polylysine chlorin e6 conjugates. *Photochem Photobiol* **65**: 723–729
Spikes JE (1986) Yearly review: phthalocyanines as photosensitizers in biological systems and for the photodynamic therapy of tumors. *Photochem Photobiol* **43**: 691–699
Svaasand O (1984) Optical dosimetry for direct and interstitial radiation therapy of malignant tumors. In: *Porphyrin Localization and Treatment of Tumours*, Doiron DR and Gomer CJ (eds), pp. 91–114. Alan R. Liss: New York
Tada HR, Shibo O, Kuroshima K, Koyama M and Tsukamoto K (1986) An improved colorimetric assay for interleukin 2. *J Immunol Methods* **93**: 157–165
Tralau CJ, Young AR, Walker NP, Vernon DI, Macrobert AJ, Brown SB and Brown SG (1989) Mouse skin photosensitivity with dihaematoporphyrin ether (DHE) and aluminium sulphonated phthalocyanine (AISPc): a comparative study. *Photochem Photobiol* **49**: 305–312
Tsuruo T, Yamori T, Naganuma K, Tsukagoshi S and Sakurai Y (1983) Characterization of metastatic clones derived from a metastatic variant of mouse colon adenocarcinoma 26. *Cancer Res* **43**: 5347–5442
van Lier JE (1990) Phthalocyanines as sensitizers for PDT of cancer. In: *Photodynamic Therapy of Neoplastic Diseases*, Kessel D (ed), pp. 279–290. CRC Press: Boca Raton, FL
Woodburn K, Chang CK, Lee B, Henderson B and Kessel D (1994) Biodistribution and PDT efficacy of a ketochlorin photosensitizer as a function of the delivery vehicle. *Photochem Photobiol* **60**: 154–159
Wulff G and Röhle G (1974) Results and problems of O-glycoside synthesis. *Angewandte Chemie* **13**: 157–170
Yamaoka T, Tabata Y and Ikada Y (1994) Distribution and tissue uptake of poly(ethylene glycol) with different molecular weights after intravenous administration to mice. *J Pharmaceut Sci* **83**: 601–606
Zharkova NN, Kozlov DN, Smirnov VV, Sokolov VV, Chissov VI, Filonenko EV, Sukhin DG, Galpern MG and Vorozhtsov GN (1994) *SPIE – Photodynamic Therapy of Cancer II* **2325**: 400

*© 1999 Cancer Research Campaign*
